# Reproductive strategies of the coral *Turbinaria reniformis* in the northern Gulf of Aqaba (Red Sea)

**DOI:** 10.1038/srep42670

**Published:** 2017-02-14

**Authors:** Hanna Rapuano, Itzchak Brickner, Tom Shlesinger, Efrat Meroz-Fine, Raz Tamir, Yossi Loya

**Affiliations:** 1 Department of Zoology, The George S. Wise Faculty of Life Sciences, Tel-Aviv University, Tel-Aviv 69978, Israel; 2 The Interuniversity Institute for Marine Sciences, P.O. Box 469, Eilat 8810369, Israel

## Abstract

Here we describe for the first time the reproductive biology of the scleractinian coral *Turbinaria reniformis* studied during three years at the coral reefs of Eilat and Aqaba. We also investigated the possibility of sex change in individually tagged colonies followed over a period of 12 years. *T. reniformis* was found to be a stable gonochorist (no detected sex change) that reproduces by broadcast spawning 5–6 nights after the full moon of June and July. Spawning was highly synchronized between individuals in the field and in the lab. Reproduction of *T. reniformis* is temporally isolated from the times at which most other corals reproduce in Eilat. Its relatively long reproductive cycle compared to other hermaphroditic corals may be due to the high reproductive effort associated with the production of eggs by gonochoristic females. Sex ratio in both the Aqaba and Eilat coral populations deviated significantly from a 1:1 ratio. The larger number of males than of females may provide a compensation for sperm limitation due to its dilution in the water column. We posit that such sex allocation would facilitate adaptation within gonochoristic species by increasing fertilization success in low density populations, constituting a phenomenon possibly regulated by chemical communication.

Research on scleractinian coral reproduction is a prerequisite for the study of other life-history strategies, the ecology and persistence of populations and communities, and for the management and preservation of the reef^1^,^2^,^3^. The evolution of our understanding of reproductive strategies since the early twentieth century has emphasized the importance of detailed descriptions of reproductive biology from individual species within the context of distributional gradients, and varying environmental conditions and habitats. The paradigm shift from internal fertilization as a commonly accepted rule^1^ to a later understanding of external fertilization through broadcast-spawning as the dominant mode of reproduction^2^ is one such example. Varying degrees of interspecific synchrony reported in coral reproduction among different regions, from the mass spawning on the Great Barrier Reef^4^,^5^,^6^ to the temporal reproductive isolation described in the northern Gulf of Eilat, Red Sea^7^,^8^ (but see Hanafy *et al*.^9^; Bouwmeester *et al*.^10^) have likewise expanded our perspective on reproductive patterns among these unique species.

As sessile organisms, corals face different challenges to reproduction than those encountered by free-living organisms that enjoy the benefit of social interactions. While most organisms reproduce as gonochorists (dioecy) that partition female and male functions between individuals, the majority of scleractinian corals reproduce as hermaphrodites, in which individuals express both male and female functions^2^,^11^. Simultaneous hermaphrodites produce both female and male gonads within the same reproductive cycle, while sequential hermaphrodites change from one sex to the other over the course of their lifetime^12^. In plants for example, the frequency of hermaphroditism is associated with their lack of mobility, and may be an adaptation to a sessile lifestyle by increasing the probability of finding each sex in a given area^13^. Thus, only a little over one third of the stony corals studied to date reproduce as gonochorists while most are hermaphrodites^1^,^2^,^11^,^14^. The fertilization of eggs and development of larva may be internal (brooders) or external (broadcast spawners)^1^.

The high variety of their reproductive strategies makes corals stimulating case studies for examining sex allocation theory. The theory seeks to explain the different ways in which species and individuals apportion resources towards each sex in order to maximize fitness^15^,^16^. Sex ratio, i.e. the number of females and males in a population is an expression of sex allocation in gonochorists. In hermaphrodites resources are allocated towards female and male functions in an individual to varying degrees. In sequential hermaphrodites sex allocation theory examines the point in life at which the organism changes from one sex to the other. Sex allocation is often discussed in context with environmental conditions^17^,^18^. One well known example for this is the Trivers and Willard^19^ hypothesis, where conditional sex allocation is predicted if parental quality influences males and females differently. The theory has seen marked success in predicting facultative sex allocation especially in sex changers and hymenopterans that can control the sex of their offspring in response to local environmental conditions^16^,^18^,^20^,^21^. Challenges faced in testing these theories lie, among others, in the difficulty of measuring the differences in cost of reproduction i.e., the trade-off between reproductive effort and survivorship between the sexes, that are often correlated with biased sex ratios^17^. Comparisons of gonad volume to somatic tissue ratios (as an approximation for productive effort) between the sexes^22^,^23^ may lead to erroneous conclusions in assuming equal metabolic cost and resources for spermatogenesis and oogenesis per unit gonad volume^1^. The theory is further complicated by unknown sex determination systems (i.e. chromosomal or environmentally determined) and the degree to which they constrain control of sex ratios^16^,^18^,^20^.

Kramarsky-Winter and Loya^24^ and later Loya and Sakai^21^ demonstrated various predictions of sex allocation theory, such as the size advantage hypothesis (SAH)^13^,^16^ on solitary coral fungiid species. The hypothesis predicts that sex change in sequential hermaphrodites will occur when a threshold body size or age is reached. At this size, reproduction is most efficient and fertility is higher for one sex over the other, making sex reversal advantageous. Another important prediction tested was that of a biased sex ratio towards the “first sex” (i.e. the sex at which individuals sexually mature)^16^,^20^,^25^. True sex change in corals was only ever observed in solitary fungiid corals^21^,^26^ over consecutive reproductive cycles, though it has been suggested that in the colonial coral *Diploastrea heliopora* polyps may switch sexes with oogenic and spermatogenic cycles occasionally overlapping^27^. The appearance of cosexual individuals (i.e., polyps and colonies with both female and male functions) within colonial gonochoristic species, although rare, suggests a transition of sexual function from one sex to the other and the potential for sex alteration within colonial species as well as solitary corals.

Very few studies have been able to resolve similar questions regarding other corals, due to the difficulty in following individuals for multiple years. This is primarily because small colonies cannot sustain repeated sampling^1^. Instead, inference is often made from correlations of sex and size under the SAH. Only seven gonochoric species of colonial corals have been repeatedly sampled over multiple consecutive reproductive cycles (i.e. *Porites cyclindrica, P. lobata* and *P. lutea*^28^,^29^; *P. australiensis*^29^; *Turbinaria mesenterina* and *Pavona cactus*^30^; and *Montastrea cavernosa*, A. Szmant, pers. comm., reviewed by Harrison and Wallace^1^) albeit only over a period of two to three years (excluding *M. cavernosa,* for which data are not available).

Here we provide the first detailed description of the reproductive biology of *Turbinaria reniformis* Bernard, 1896 in the Gulf of Aqaba/Eilat (hereafter the GOA/E), northern Red Sea with regard to sexuality, mode of reproduction, sex ratio, gametogenic development, size at earliest reproduction, fecundity, and timing of reproduction. We were particularly motivated by the question of sex change in colonial corals, for which the study was made possible by the uniquely extended monitoring period (12 years) of tagged colonies spanning 2003–2015, following a period of acute degradation^31^,^32^ and subsequent trend of recovery of the reefs at Eilat^33^. For a detailed account of historic anthropogenic perturbations resulting in changes in the coral community structure at Eilat see Loya^31^ and Loya^32^. Finally, we discuss considerations of male-female fitness trade-offs possibly motivating sex allocation in corals within the context of demographics and population distributions.

## Results

### Reproductive strategies and gonad arrangement

*Turbinaria reniformis* was found to be gonochoric (Fig. 4a,b), with all polyps within a colony belonging to a single gender. The four female colonies and five male colonies studied between the years 2003–2015 at the IUI in Eilat were reproductive throughout the study years. All nine individual colonies maintained their sex (i.e., no sex change was recorded) with no detectable occurrence of hermaphroditism. *T. reniformis* reproduces via broadcast spawning, observed to be synchronous both in the lab and in the field. Male and female gonads are enveloped within the mesenterial mesoglea in association with the mesenterial filaments (Fig. 5c). Polyps are typically structured with 12 mesenteries, though they may contain up to 16 mesenteries, all potentially capable of carrying gonads. The gonads are spread throughout the mesenteries when mature. The polyps are arranged with the aboral end of one polyp extending horizontally beneath its proximal predecessor. Willis^30^ described a similar “L”-shaped growth pattern in *Turbinaria mesenterina*.

### Seasonal trends in gametogenesis

*Turbinaria reniformis* reproduces annually with spermatogenesis succeeding oogenesis. The earliest conspicuously discernible female gametes (stage I) appeared in September 2014 (Fig. 3a), indicating the onset of gametogenesis, and in 2015 were first seen in August. Thus, the oogenic cycle is presumed to last between 11–12 months. However, stage I was also observed as early as May and June 2014 and May 2015 in negligible numbers (only one or two oocytes).

During the 2014–2015 cycles oocytes increased from a monthly mean diameter of 74 ± 18 μm (mean ± SD, n = 14) in September 2014 to a maximum of 480 ± 114 μm (mean ± SD, n = 30) in June 2015 (Fig. 2a). The most noticeable growth in oocytes appeared between September and March, with a monthly mean diameter of 384 ± 55 μm (mean ± SD, n = 40), after which maturation proceeded more gradually as oocytes become more crowded. Mean monthly oocyte diameters from 2003–2009 samples were closely associated to the values measured in 2015 (Fig. 2a), and corresponded to the general trend of oocyte maturation in 2014–2015. Measurements from July and August in particular, appeared as the most variable throughout all the years of observation (Fig. 2a and Supplementary Table S1). This variation agrees with the variety of stages found present in August 2015 (17% of stage I, 5% of stage II, 10% of stage III and 67% of stage IV; Fig. 3a).

Stage I oocytes (Fig. 5a) with an average size of 69 ± 35 (mean ± SD, n = 15) comprised approximately 80% of those found in September 2014 (Fig. 3a). Stage II oocytes (Fig. 5c), averaging 177 ± 91 (mean ± SD, n = 32) in diameter, were first seen in September 2014. Comprising approximately 50% of the oocytes by October 2014, stage II gradually decreased, although persisting throughout the reproductive season in small numbers. Their presence throughout the year suggests the presence of stage I as well, although the earlier stages are more difficult to discern. Stage III (Fig. 5c), with an average diameter of 320 ± 67 μm (mean ± SD, n = 30) became a majority by March 2015 (76%). Stage IV oocytes (Fig. 5b) measured 475 ± 108 μm (mean ± SD, n = 77) in average diameter and were first observed as early as March 2015, comprising the predominant stage from June to August 2015 (60–68%). In July 2014 only stage IV was observed. By August 2015, stage I was observed developing concomitantly alongside stages III and IV, marking the beginning of the subsequent cycle.

The earliest stage of male reproduction (Stage I) was first observed in November 2014 (Figs 3b and 4d). Stage I remained abundant within polyps till May. Stage II (Fig. 5e) was present alongside stage I, beginning in November through to June 2015, with the highest abundance in May 2015 (57%; Fig. 3b). Stage III (Fig. 5f) spermaries increased rapidly from 28% in May 2015 to 70% in June. By July the majority of the spermaries had matured into stage IV (90%; Figs 3b and 4g). A decrease in spermary size was evident between July and August in 2014 and 2015: from 118 ± 55 μm (mean ± SD, n = 47) in July to 81 ± 16 μm (mean ± SD, n = 20) in August 2014, and from 163 ± 76 μm (mean ± SD, n = 30) in July to 95 ± 34 μm (mean ± SD, n = 39) in August 2015 (Fig. 2b).

The proportion of male and female reproductive colonies also decreased between July and August 2015, from 100% to 75% (Fig. 2a,b), suggesting an initial release of the largest gametes between July and August 2015. Mature (stage IV) oocytes and spermaries were absent by August 2014 and early September 2015 (Fig. 3a,b) indicating that the remaining gametes had spawned in August. In July 2006 highest mean oocyte diameter was 430 ± 151 μm (mean ± SD, n = 13), and by the end of August only one colony still was reproductive, with stage I oocytes measuring 115 ± 17 μm in diameter (mean ± SD, n = 8; Supplementary Table S1). This provides further indication of a release between July and August. At the very end of July 2008 and 2009 the majority of the corals (3 out of 4 in both years) were non-reproductive (Supplementary Table S1), with the remaining samples still bearing mature oocytes.

Interestingly, while no oocytes were detected in August 2014 (Fig. 3a,b) two males out of five appeared to still contain mature gametes (Fig. 2b). It is thus likely that partial spawning occurs at the colony level and that we had missed sampling the reproductive areas in the female colonies during that month.

The start of the gametogenic cycle, beginning with the oogenic cycle, followed the highest annual mean sea surface temperature (SST) in August and proceeded with the early developmental stages as temperatures declined (Fig. 2). Final oocyte and spermary maturation between April and August 2015 followed rising water temperatures.

### Spawning periodicity

Released gametes were observed in isolated *T. reniformis* fragments in mid-July 2014 (five and six days after the full moon) in Eilat. No reproduction was observed in June 2015 in isolated fragments kept in running sea-water tables or *in situ*. In 2015, eggs were observed from one isolated fragment from Eilat in early July (two days after the full moon) between 20:00 and 23:00. Mature oocytes were still present in histological sections of fragments collected on the date of the full moon in July. Likewise, gametes were found in containers with isolated female and male fragments from Aqaba early in August, five and six days after the full moon. In 2016 spawning was observed *in situ* at the southern end of the Eilat Coral Nature Reserve (Fig. 4a,b, Supplementary Video S1) for three nights, beginning five days after the full moon in June, between 20:30–21:00. On the first night only eight males had spawned, while others around them remained inactive. On the following nights both females and males (a total of 10–20 colonies each night) were spawning within minutes of each other. During the three nights where *T. reniformis* colonies were observed spawning, no other scleractinian coral species were seen spawning at the same time. Only two species were observed spawning on the same nights as *T. reniformis*. Colonies of *Lobophyllia sp.* were seen spawning on the first night, ending several minutes prior to *T. reniformis* spawning. Colonies of *Platygyra lamellina* spawned on all three nights, but *ca.* 1.5 hours before *T. reniformis*. No colonies were seen spawning in July and August 2016. The eggs released were brownish with moderate positive buoyancy (Fig. 4a).

### Fecundity

Female colonies of *T. reniformis* in Eilat produced an average of 69 ± 47 (mean of colony averages ± SD, n = 4 colonies) oocytes per polyp in 2014, and 207 ± 107 (mean of colony averages ± SD, n = 4 colonies) oocytes per square cm of tissue.

### Sex ratio and sizes of male and female colonies

A study of the distribution of *T .reniformis* sexes at the Interuniversity Institute for Marine Sciences site in Eilat (IUI) and the Marine Science Station site in Aqaba (MSS) revealed no significant differences (at the 0.05 significance level) in the sex ratio of females and males between the two locations (*p* = 0.772, two-sided Fisher’s exact test). At both locations the sex ratios differed significantly from 1:1, where male colonies significantly outnumbered female colonies (Table 1) i.e., in Eilat (8 females, 20 males, Chi-square test, **χ**^**2**^ = 5.14, *df* = 1, *p* = 0.02) and in Aqaba (8 females, 27 males, Chi-square test, **χ**^**2**^ = 10.314, *df* = 1, *p* = 0.001).

The smallest male found had a mean geometric diameter of 15 cm, and the smallest female had a mean geometric diameter of 21 cm (Fig. 6). Smaller colonies did not contain gonads. The largest colony found (148 cm mean geometric diameter) was sterile, possibly due to senescence^34^, and therefore, was not included in the analysis of female and male sizes.

A two-way ANOVA test comparing female and male colony sizes (mean geometric diameters; Fig. 6), with sex and location (Aqaba and Eilat) as factors, indicated no significant difference (at the 0.05 significance level) between female and male sizes in Aqaba (8 females and 27 males) and in Eilat (8 females and 20 males), F (1, 52) = 0.109, *p* = 0.743. Additionally, no significant difference was found between Aqaba and Eilat locations, F (1, 52) = 0.678, *p* = 0.418, and no significant interaction was found between sex and location (*p* = 0.418).

## Discussion

This is the first comprehensive study of the reproductive biology of *Turbinaria reniformis,* a gonochoric (dioecious) broadcast spawner in the northern Red Sea. Data collected throughout the study (2003–2009 and 2014–2015) indicate a clear periodic pattern of annual reproduction, with a cycle lasting 11–12 months. However, evidence of the first recognizable stages as early as May and June of 2014 (although negligible in numbers) may suggest an even longer cycle.

The reproductive strategies of *T. reniformis* in this study agree with the only two reports on this species. The first was by Willis *et al*.^5^ in Australia where one colony was observed spawning in a flow-through aquaria system six days past the full moon in November, and was classified as gonochoric. The second was an account of a single colony spawning in an aquarium system hosting Indo-Pacific corals five days after the full moon in June, at approximately 19:30 hours^35^. Although data from *ex-situ* spawning events from one colony in each report are available, the timing within seasons and lunar cycle at which they spawned in all reports seemingly correspond. The spawning in Australia and in the current study in the northern GOA/E preceded average annual peak water temperatures by one to two months (*sensu* Willis *et al*.^5^) and in all three studies occurred 5–7 days after the full moon. Though *T. reniformis* might spawn earlier on in the season in Australia than in the Red Sea, temporal diversity in reproduction is not uncommon over a distribution range and may be a result of adaptation to local environments^11^. Nevertheless, mode of reproduction and sexuality appear conservative among these allopatric populations.

Common reproductive traits often span genera and even families^1^,^14^, with the *Dendrophylliidae* family being predominately gonochoric^23^,^36^. All available reports within the genera show *Turbinaria* to be uniform in both reproductive mode and sexuality: *T. frondens, T. mesenterina, T. stellulata* and *T. reniformis* are gonochoric spawners and *T. bifrons, T. peltata,* and *T. radicalis* are broadcast spawners, with sexuality (hermaphroditism or gonochorism) unknown^5^,^10^,^14^,^30^.

While other *Turbinaria* species reproduce uniquely in austral winter^30^, *T. reniformis* in Eilat apparently conforms to the reproductive seasonality in the northern GOA/E, where stony corals generally reproduce during the summer^7^,^11^,^37^. Moreover, breeding in *T. reniformis* seems interspecifically asynchronous in month or lunar phase to other species’ reproduction periods in Eilat. *Dipsastraea favus* and *Galaxea fascicularis* are the only species whose estimated breeding time reported in literature may partially overlap with *T. reniformis*^7^. Nevertheless, out of dozens of species occurring on the same reef, only *Platygyra lamellina* and *Lobophyllia sp.* were observed spawning each on one or all of the three nights that *T. reniformis* was seen spawning in the field in 2016, albeit all at different hours. The asynchronous spawning among the most abundant northern GOA/E corals, termed “temporal reproductive isolation”, might enable minimizing potential larval competition for available settlement substrate, as well as possible hybridization^7^,^8^.

Various environmental cues are attributed to reproductive synchronization within coral species^1^,^2^ with changes in water temperatures still the most common factor correlated with gamete development and spawning^38^,^39^. Final female and male gamete maturation of *T. reniformis* indeed coincides with the rising temperatures in the GOA/E (Fig. 2). A similar pattern is observed in the congener *T. mesenterina*, where oocytes grow rapidly during sharp rising temperatures in Nelly Bay at Magnetic Island^30^. Spawning beginning before reaching annual SST highs in August may provide an escape from the thermal stress shown to affect early life stages in some species^40^. While rising temperatures might facilitate the necessary physiological requirements for gamete maturation^30^, lunar cycles are believed to act as a cue for initiation of reproduction after maturation is reached^6^. According to observations from isolated fragments in 2014–2015 and field observations in 2016, *T. reniformis* reproduced during the last quarter moon, like many scleractinian corals (see Harrison and Wallace^1^; Harrison^2^), with notable synchrony in the lunar phase (five to six days after the full moon) during the three years of observation (2014–2016). Females and males also appeared highly synchronized in time of reproduction on the second and third nights of observation in 2016 (Supplementary Video S1). The initial male release observed on the first night in 2016 was ambiguous and additional observations are needed in order to confirm whether a pattern exists.

Evidence from spawning observed in July 2015 and inference from spawning between August and September from histological sections in 2015 suggest that *T. reniformis* experience protracted spawning (i.e., multiple spawning events over consecutive lunar cycles) over the course of several years. The occurrence of histological samples devoid of female and male gonads between July and August of 2014 and 2015 and in July 2008 and 2009 suggests an initial release prior to August. Further evidence of this strategy is found in the gradual decline in average spermary sizes between June and September in 2014 and 2015 (Fig. 2a,b), and may also explain the concomitant existence of various stages of development, especially among female colonies of *T. reniformis*, over much of the reproductive cycle (Fig. 3a).

Protracted spawning is not uncommon among stony corals even though they exhibit remarkable synchrony in breeding^41^, and may be an attempt to increase chances of larval survival by not “putting all of the eggs in one basket”^11^. A similar spawning pattern has been reported in the congener, *T. mesenterina*, in which a gradual decline in oocyte numbers over two to three months was reported^30^,^42^. Similarly, *Acropora tenella* from the mesophotic reef in Okinawa, Japan, exhibits an extended spawning over two consecutive months^43^, as do various other shallow populations of *Acropora* spp.^44^. Successive multiple spawning events have been estimated for various species in Eilat as well (e.g. *Galaxea fascicularis, Astreopora myriophthalma,* and *Pocillopora verrucosa*^7^) and may also contribute to further temporal reproductive isolation.

The absence of gametes in histological sections in August 2014 (Fig. 3) seemingly corresponds to the absence of spawning observed in August 2016. This period of quiescence between cycles^1^ may occur in alternating years in order to maintain seasonality (*sensu* Willis *et al*.^5^, who explain the shift in the months of spawning on the Great Barrier Reef in light of the yearly variation in the calendar date of the full moon). The comparatively late start of oogenesis observed in September 2014 coincides with the later spawning inferred from histology in August 2015 compared to spawning in 2014 and 2016, occurring as early as June.

The comparatively long oogenic cycle experienced by *T. renifomis* and its congener, *T. mesenterina*^30^, along with various other broadcast spawning gonochorists (e.g. *Astrangia lajollaensis*^45^; *Paracyathus stearnsii*^46^; *Plesiastrea versipora*^38^) may be connected to a higher energetic demand placed on gonochoric individuals producing only eggs compared to their hermaphroditic counterparts. In support, Loya and Sakai^21^ reasonably posited that partitioning of resources towards females may be greater based on typically longer oogenic cycles compared to spermatogenic cycles in most corals. A lengthy cycle also appears to be characteristic of the genus^30^.

Optimal sex allocation within a population reproducing sexually is commonly thought to revolve around a 1:1 ratio of females to males^15^,^47^ provided that males and females are equally costly to produce. Many populations of gonochoric corals conform to this pattern^1^,^23^,^38^,^48^. Nevertheless, both the Eilat and Aqaba populations of *T. reniformis* exhibited male-biased sex ratios. Sex allocation theory predicts a bias in sex ratio toward the first sex (the sex at which individuals mature sexually) in sex-changing species^16^,^49^,^50^. This is probably not the case in the studied populations, as no evidence of sex change was detected in the nine individual colonies from Eilat throughout an extensive observation period. The lack of sexual dimorphism in male and female sizes within both populations strongly supports this assessment (Fig. 6).

One frequent explanation for a skewed sex ratio is that of a high measure of asexual reproduction, therefore favoring one genotype and thereby sex^28^,^29^. However, this is probably not the reason for the bias observed in this study, since throughout the whole study no broken fragments were ever observed around colonies of this species, as can often be seen on the reef in branching corals after a storm. Furthermore, fragments from the colonies we returned to the reef never survived (HR, pers. obs.).

Local gamete competition for fertilization can have vast implications for sex allocation in the sea^51^. Studies of plants and of other semi-sedentary marine animals have shown diversity in reproductive success based on the relative abundance of each sex^52^,^53^,^54^. The limitation of one sex may indeed drive an adjustment of the sex ratio, based on unique life histories. For example, a female bias in various brooding stony and soft corals^55^,^56^ has been explained by a limitation in brooding space (where only females brood)^55^. A male bias might also be favored in populations where sperm is similarly a limiting factor.

Fertilization in sessile free-spawners can be critically challenged by the dilution of sperm in seawater^57^,^58^ coupled with low population density^57^,^59^. Hence, increased male allocation in *T. reniformis* may act to counter sperm dilution under low population density, much like some asteroids that are known to increase sperm production when individuals are far apart^60^. It is difficult at this stage to say whether population densities in Eilat and Aqaba have become a challenge to successful fertilization. However, compared to the abundance of the congener, *T. mesenterina*, at Magnetic Island, as evident from the average number of colonies in transects (1 m^−1 ^30), the Eilat population appears more sparse, as reported by the National Monitoring Program (NMP) in 2014 (0.3 m^−1 ^61; Supplementary Fig. S1). This could explain why the GBR population of *T. mesenterina* could “afford” an equal sex ratio (1:1) at higher population densities.

Parallels drawn previously between corals and plant reproduction^21^,^51^ indicate pollen limitation in plants as an appropriate comparison to sperm limitation in marine free-spawners. McCauley and Taylor^62^ modeled an alteration of population structure (i.e. sex ratio) in gynodioecious plants (a reproductive strategy in which female individuals coexist with hermaphroditic individuals in the population) as a function of sex-specific fitness when pollen limits fertility. Increased pollen production has also been connected to decreased numbers of males in plant species^63^. Similar concepts may apply to variations in sex ratios based on population structure in marine gonochoristic species.

Overcoming sperm dilution may be the ultimate factor of a male-biased sex ratio, though it does not explain the mechanism regulating the skew. One possibility would be a system of density-dependent chemical communication in a population and between conspecifics, allowing an individual to sense the potential nearness or abundance of the opposite sex, or lack thereof. For example, within a sperm-limited environment an individual may become female due to a strong male signal, or male in the absence of a signal. Presumably, sex steroids, shown to regulate gametogenesis in scleractinian corals (e.g. *Oculina patagonica*^64^), could act similarly to sex pheromones shown to serve as communication in crustacea and various fish^65^.

Such facultative sex allocation would depend on the mechanism of sex determination, which is to date largely unknown in corals. Generally, sex may be determined genetically (i.e. chromosomally) or may be conditional on external cues (environmental) and determined later in development^66^. Loya and Sakai^21^ suggested that the environmental conditions may have some influence on sex change in the corals in their study, by favoring the more resource-demanding sex under favorable environmental conditions. Nevertheless, expression of a single sex throughout a lifetime suggests a deterministic genotypic mechanism^67^ which is often perceived as a constraint on control of sex ratios^18^. Though mounting evidence is pointing to adaptive sex-ratio adjustments, even in mammals and birds with genetic sex determination^18^, sex determination in *T. reniformis* could possibly also be subject to external cues, even though sex is not labile. Infrequent occurrences of hermaphroditism in various stable gonochorists (see Guest *et al*.^27^) suggests that gonochorists may have the genetic potential for both sexes. Evidence among other cnidarians suggests a combination of genetic and environmental sex determination^68^,^69^ that could allow for the influence of conspecifics on sex determination in *T. reniformis.*

Sequential and simultaneous hermaphrodites may provide functional adaptations to the same limitations of sperm using different strategies: low population densities are addressed by simultaneous hermaphrodites through an increased probability of finding each sex in a given environment and area^11^,^13^,^27^, while dynamic adjustment of sex ratios by sequential hermaphrodites could potentially counter sperm limitation. Such examples of male-biased populations of sex changing fungiid corals^21^ may benefit from such a skew.

The effects of environmental resource limitation on the differential cost of reproduction between sexes are often attributed to deviations from an equal sex ratio^16^,^17^,^70^ and may also factor into the skew observed in the two studied populations. Loya and Sakai^21^ suggested that resource allocation in fungiid corals may be a flexible response to local environmental conditions by changing sex to the more energetically costly sex under favorable conditions or to the least expensive sex under stressful conditions. Thus, if sex were labile in *T. reniformis,* we would expect sex change to occur in an environment subject to disturbances or substantial change. However, all individually tagged colonies monitored throughout 2003–2015 maintained their sex despite the environmental changes experienced by reefs at Eilat from the degradation observed between 1969 and 2004^32^ to recent evidence of gradual recovery observed in 2015^33^.

In their review, Harrison and Wallace^1^ compared a male-dominated population of *Porites porites* on a polluted reef in Barbados to equal sex ratios on nearby less polluted reefs studied by Tomascik and Sander^71^, in connection with sex allocation theories, predicting a shift towards maleness in declining environments^72^,^73^. Nevertheless, the effect of the environment driving a male skew in such cases may alternatively lie in increased sperm limitation caused by a limitation of the population through scarce resources or perturbations. Cost of reproduction alone, therefore, may not be enough to account for unequal sex allocation.

In conclusion, a lower sex ratio bias is predicted for *T. reniformis* populations of greater densities and abundance. We posit that evaluating sex allocation in light of local distribution patterns (abundance, density, and distance between males and females) and comparing them with allopatric populations, might provide a relatively testable approach to understanding anomalous sex ratios. Fertilization rates should also be considered and tested in context with biased sex ratios. While the ultimate factors of a male bias and their effect on female and male fitness have been discussed, the mechanism by which this bias is facilitated remains a mystery. Future research on sex determination in corals is crucial for understanding sex allocation among reproductive strategies.

## Materials and Methods

### Study sites and sampling

**Figure 1:**
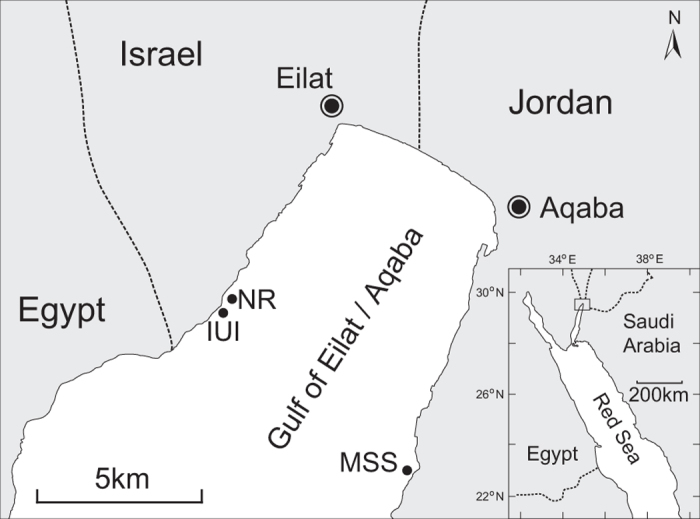
Map of the study sites in the northern Gulf of Aqaba/Eilat, Red Sea. NR indicates the Eilat Coral Nature Reserve, IUI the Interuniversity Institute of Marine Sciences in Eilat, and MSS the Marine Science Station in Aqaba. The map was drawn using Adobe Illustrator CS 6.

**Figure 2:**
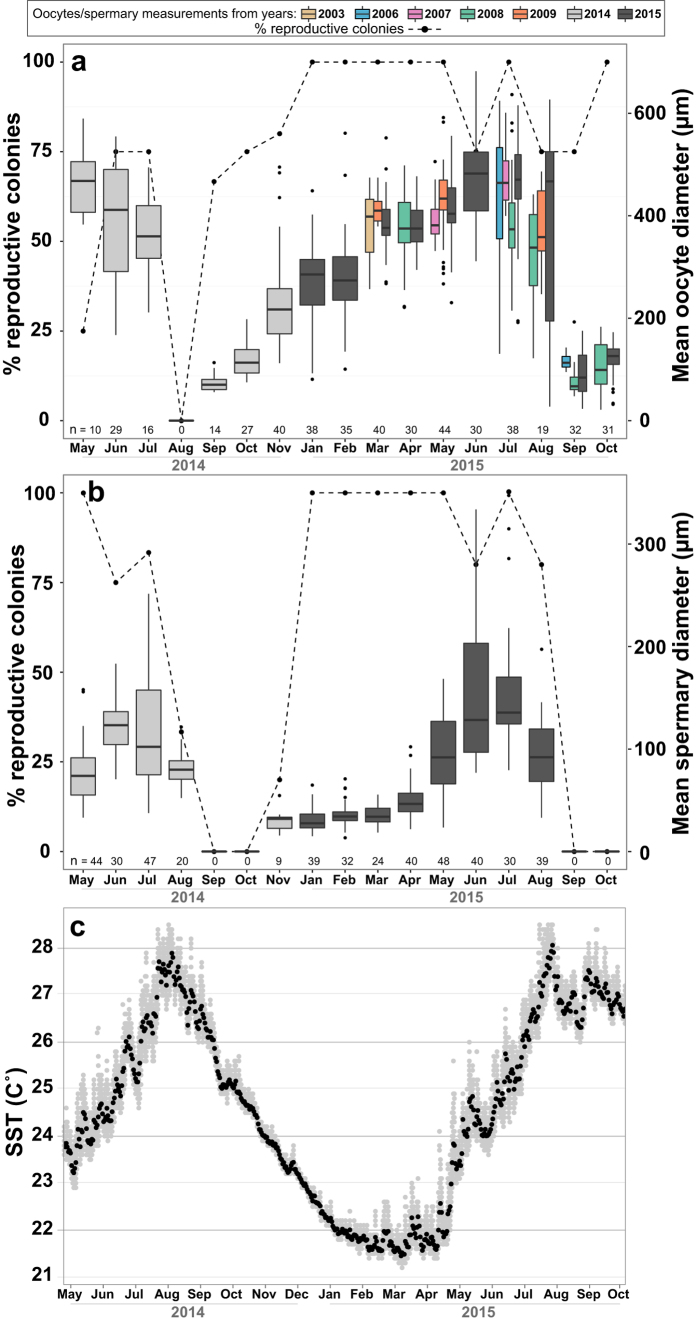
Seasonal patterns of gametogenesis of *Turbinaria reniformis* from Eilat (Gulf of Aqaba). Growth in mean oocyte (**a**) and spermary (**b)** diameter (μm), and percentage of reproductive colonies (n = 3–5 colonies and one female in May 2014 [**a**]) measured from histological sections. Gray boxes represent measurements during 2014-2015. Colored boxes represent measurements from 2003, 2006–2009 plotted alongside the nearest dates in 2015. Error bars represent SD, and n = number of colonies sampled. Box limits (**a,b**) represent 25^th^ and 75^th^ percentiles, whiskers span the upper and lower limits of the data and black dots represent possible outliers. Horizontal lines within boxplots represent the medians and n = number of oocytes (**a**) or spermaries (**b**) measured per month in 2014–2015. The number of oocytes measured from 2003–2009 are available in Supplementary Table S1. (**c**) Daily SST (°C) at hourly intervals throughout sampling in 2014–2015 measured at the IUI site and represented by the gray dots. Average daily measurements are represented by the black dots.

**Figure 3:**
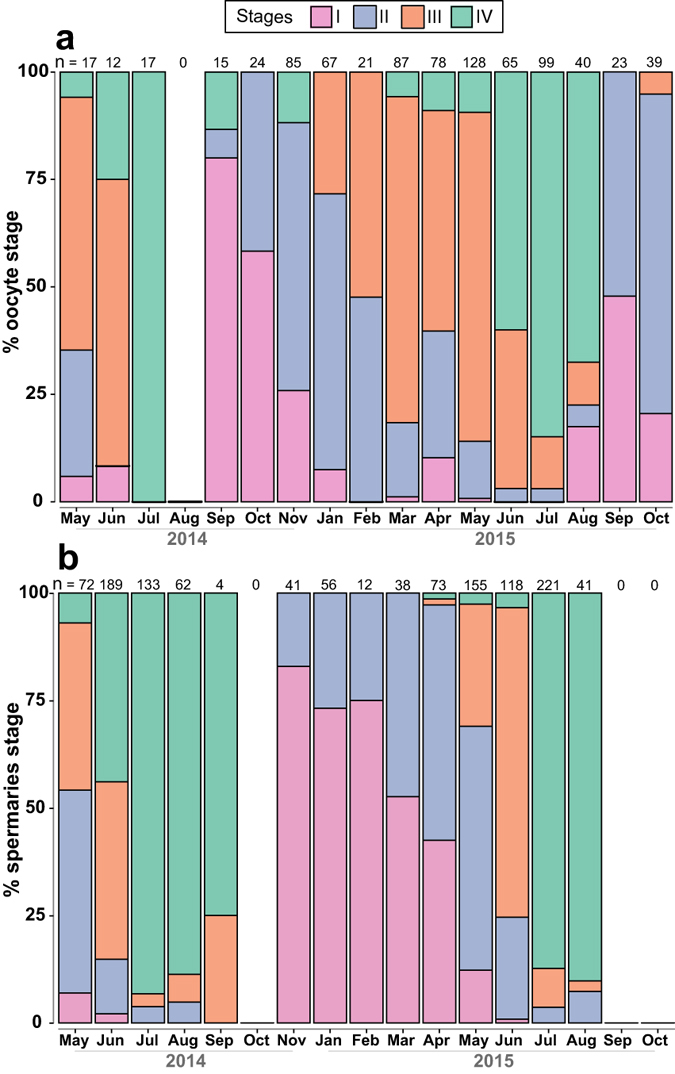
Temporal changes in oocyte and spermary development in Turbinaria reniformis from Eilat (Gulf of Aqaba). Monthly frequencies (%) of female (**a**) and male (**b)** developmental stages (I-IV) are indicated in color and were compiled from monthly samples (3-5 colonies and one female colony in May 2014). n = number of oocytes (**a**) and spermaries (**b)** evaluated from histological sections.

**Figure 4:**
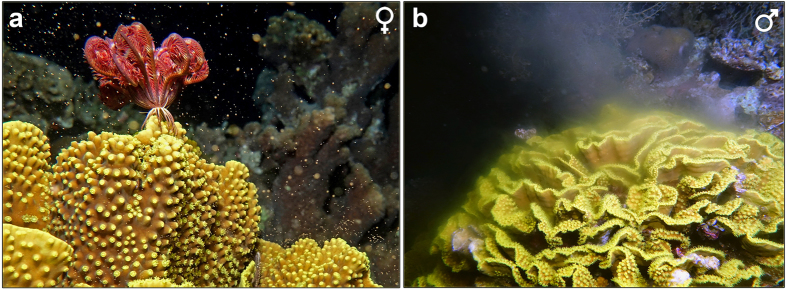
Spawning of female (**a**) and male (**b**) *Turbinaria reniformis* colonies observed at the Eilat Coral Nature Reserve in July 2016.

**Figure 5:**
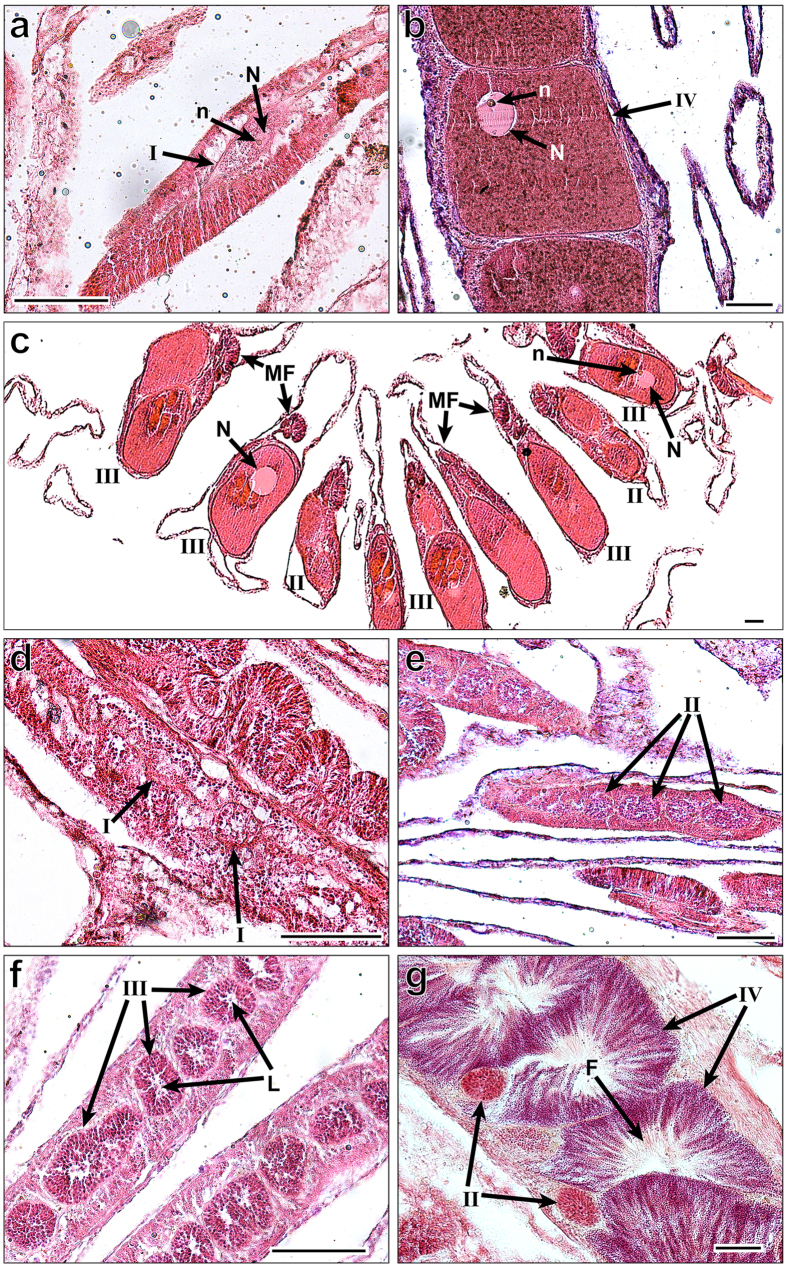
Histology of Female (**a–c**) and Male (**d–g**) developmental stages of *Turbinaria reniformis* from Eilat (Gulf of Aqaba). Scale bars indicate 100 μm. Female (**a,b,c**): I first descernable oocytes; II–III developemental stage II and III oocytes; IV mature oocytes stain a darker red and nuclei have migrated to the periphery of the oocyte; N nucleus; n nucleolus; MF mesentarial filaments. Males (**d,e,f,g**): I clusters of interstitial cells; II-IV developmental stages II–IV of spermaries. L lumen in the center of spermaries; F visible flagella.

**Figure 6:**
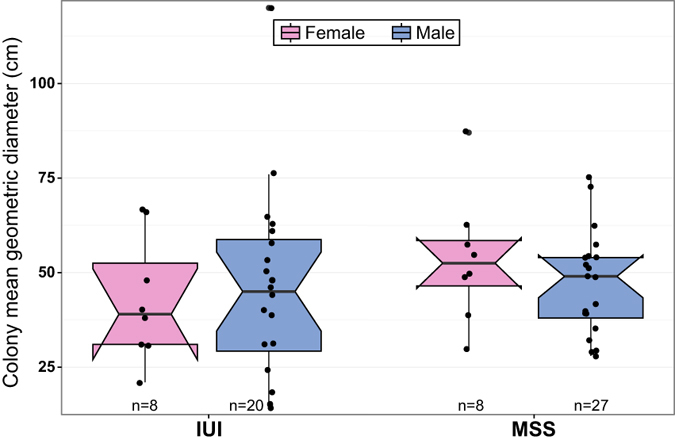
A comparison of female and male colony sizes (mean geometric diameter in cm) of *Turbinaria reniformis* at the Aqaba and Eilat sites. Box limits represent the 25^th^ and 75^th^ percentiles; the horizontal lines within the boxes show the median and whiskers the upper and lower limits of the data. The notches indicate the 95% confidence interval around the median +/−1.57 × IQR/sqrt (n). Data points are indicated by the black dots with possible outliers outside the whisker limits. n = number of colonies measured in each group.

All samples and measurements for this study were obtained by SCUBA diving at two sites in the northern GOA/E (Fig. 1 and Supplementary Fig. S1): in front of the Interuniversity Institute for Marine Sciences in Eilat (IUI) (29°30′N, 34°55E); and in front of the Marine Science Station in Aqaba (MSS) (29°27′N, 34° 58″E). Both sites are referred to synonymously by their respective cities. Additional field observations were conducted at the southern end of the Eilat Coral Nature Reserve (NR; Fig. 1), approximately 250 meters north of the IUI site.

### Sampling regime, processing, and histological studies

In order to examine the reproductive cycle, nine large *Turbinaria reniformis* colonies (four females and five males between 39–75 cm mean geometric diameter; see Loya^74^), found between 4–10 m depth at the IUI site, were tagged and re-sampled sporadically from 2003–2009 during March-October (dates of sampling are available in Supplementary Table S1). Sequential monthly sampling of the same colonies was conducted between May 2014 and October 2015, excluding December. Fragments (3–4 cm) were broken off from healthy leaves that had not previously been sampled, using a cutter. The samples were fixed overnight in 4% seawater formalin within an hour of collection, rinsed in running tap water, and preserved in 70% ethanol. Fragments were decalcified in 25% formic acid buffered with citrate (equal parts of 20% tri-sodium citrate in distilled water and 50% formic acid in distilled water). Approximately 1 cm was trimmed off the edges of the tissue in order to avoid a potential sterile zone^75^. The tissue was then dehydrated, embedded in paraffin, cross-sectioned at 7 μm, mounted on slides, and stained with Mayer’s hematoxylin and Putt’s eosin (H&E).

Histological observations were used to evaluate temporal changes in the gametogenetic cycle. The clearest slides of the three replicates per sample were examined under a Nikon Eclipse 90i microscope using NIS Elements D 3.2 software. Mean oocyte sizes per month were obtained by measuring the longest axis of all oocytes (with visible nuclei) in a slide. The ten largest oocytes per sample (or upper 30% if the number of oocytes was lower than twenty) were plotted. Measurements from 2003–2009 in March-October were plotted alongside values from the nearest sampling dates in the 2015 cycle. The longest axis of randomly selected spermaries within each polyp were also measured (5–50 per slide) in order to represent temporal development of male gametes over the spermatogenic cycle.

Female and male gonads from histological observations were classified into four stages according to Glynn *et al*.^76^. Following the system designed by Szmant-Froelich^77^, the number of each stage for female and male gonads (oocytes or spermaries) was recorded for five mesenteries within each sample. Counts of each stage were compiled from each sample for each sampling date, and the percentage of each stage was calculated from the total number of female or male gonads staged for that sampling date.

### Observations of reproduction

Preliminary observations of three to six isolated fragments (from both female and male colonies) were carried out in flow-through water tables with running seawater up to seven nights after the full moon in July 2014 and July and August 2015 in both Eilat and Aqaba. Field observations were later conducted in 2016 at the Eilat Coral Nature Reserve (Fig. 1) for three to five hours every night from early June to the end of August.

### Fecundity

**Figure 7:**
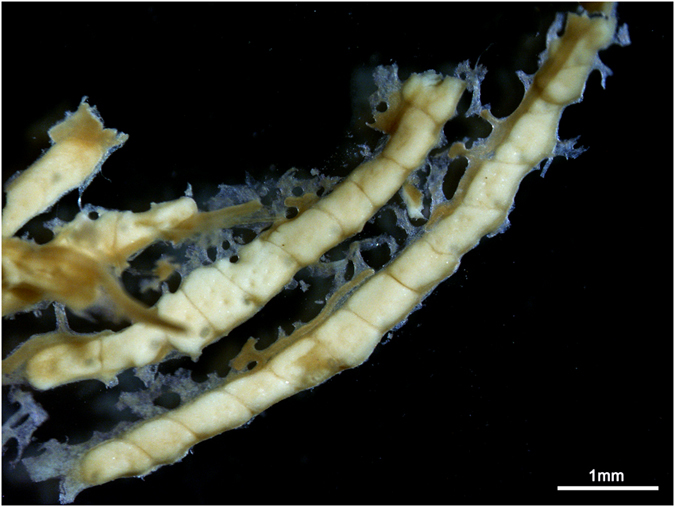
Dissected mature oocytes from *Turbinaria reniformis.*

In order to measure the fecundity of *T. reniformis*, decalcified samples from the four females in Eilat in 2014 were dissected shortly before reproduction (estimated from histology) under a stereo microscope (Nikon SMZ1500 mounted with the TV lens 055X DS Nikon Japan) using precision tweezers (Fig. 7). Oocytes were counted from five polyps for each sample. The number of oocytes per polyp was then averaged per sample. The area dissected was also measured using Nis Elements D 3.2 software so that the number of oocytes could be normalized to surface area as well as per polyp.

### Sex ratio and size distribution

For comparison of female and male sizes and sex ratios between sites, 43 and 32 colonies from Eilat and Aqaba, respectively, were randomly selected from five to ten meters depth over a distance of approximately 200 meters. All colonies were measured (circumference, width, and height) and sexed by dissection of decalcified samples under a stereo microscope. If the sex could not be clearly determined from dissection it was re-examined through histology. Mean geometric diameters of the colonies were calculated from measurements according to Loya^74^, assuming a spherical shape. Height was measured from the base of the colony to its highest point, and width was measured across the colony from the tips of the branches farthest from each other. Diameter (d) was calculated from the circumference (c) in the equation:


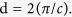


Diameter calculated from circumference served as the width in Loya’s^74^ equation:


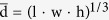


where l = length; w = width; h = height; 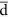 = mean geometric diameter.

### Sea-surface temperatures

Sea-surface temperatures (SST) were provided by the Israel National Monitoring Program in the GOA/E (http://www.meteo-tech.co.il/EilatYam_data/ey_observatory_pier_download_data.asp). Daily temperature measurements from the sampling dates in 2014–2015 at hourly intervals were plotted for comparison with the oogenic and spermatogenic cycles during those years.

### Analysis

Statistical analyses were performed using R statistical software R Core^78^. Fisher’s Exact test was applied in order to check for differences between the sex ratios at the Eilat and Aqaba sites. Chi-square tests were used to test for possible deviations from a 1:1 sex ratio. A two-way ANOVA was conducted on the sizes (mean geometric diameter) of colonies, with the sex and location (Eilat and Aqaba) as factors. The data on sizes of the colonies were tested and found satisfactory for requirements of normality and homogeneity of variance.

## Additional Information

**How to cite this article**: Rapuano, H. *et al*. Reproductive strategies of the coral *Turbinaria reniformis* in the northern Gulf of Aqaba (Red Sea). *Sci. Rep.* **7**, 42670; doi: 10.1038/srep42670 (2017).

**Publisher's note:** Springer Nature remains neutral with regard to jurisdictional claims in published maps and institutional affiliations.

## Supplementary Material

Supplementary Video S1

Supplementary Information

## Figures and Tables

**Table 1:** Sex ratio of *Turbinaria mesenterina* populations in Eilat (IUI) and Aqaba (MSS).

Site	n	% reproductive colonies	Number of females	Number of males	Sex ratio	χ^2^	*p*-value
IUI	32	85	8	20	2.5	5.14	*p* = 0.002
MSS	43	81	8	27	3.38	10.31	*p* = 0.001
