# Supplementary material for: Reproductive strategies of the coral Turbinaria reniformis in the northern Gulf of Aqaba (Red Sea)
Source: Sci Rep. 2017 Feb 14;7:42670. doi: 10.1038/srep42670 (PMC5307385; doi:10.1038/srep42670)
Supplement: Supplementary Information [file srep42670-s2.pdf]

Reproductive strategies of the coral *Turbinaria reniformis* in the northern Gulf of Aqaba (Red Sea)

[1]Hanna Rapuano[*], [1]Itzchak Brickner, [1]Tom Shlesinger, [2]Efrat Meroz-Fine, [1,2]Raz Tamir,[1]Yossi Loya

Supplementary information:

Location of studied *Turbinaria reniformis* colonies at the IUI site

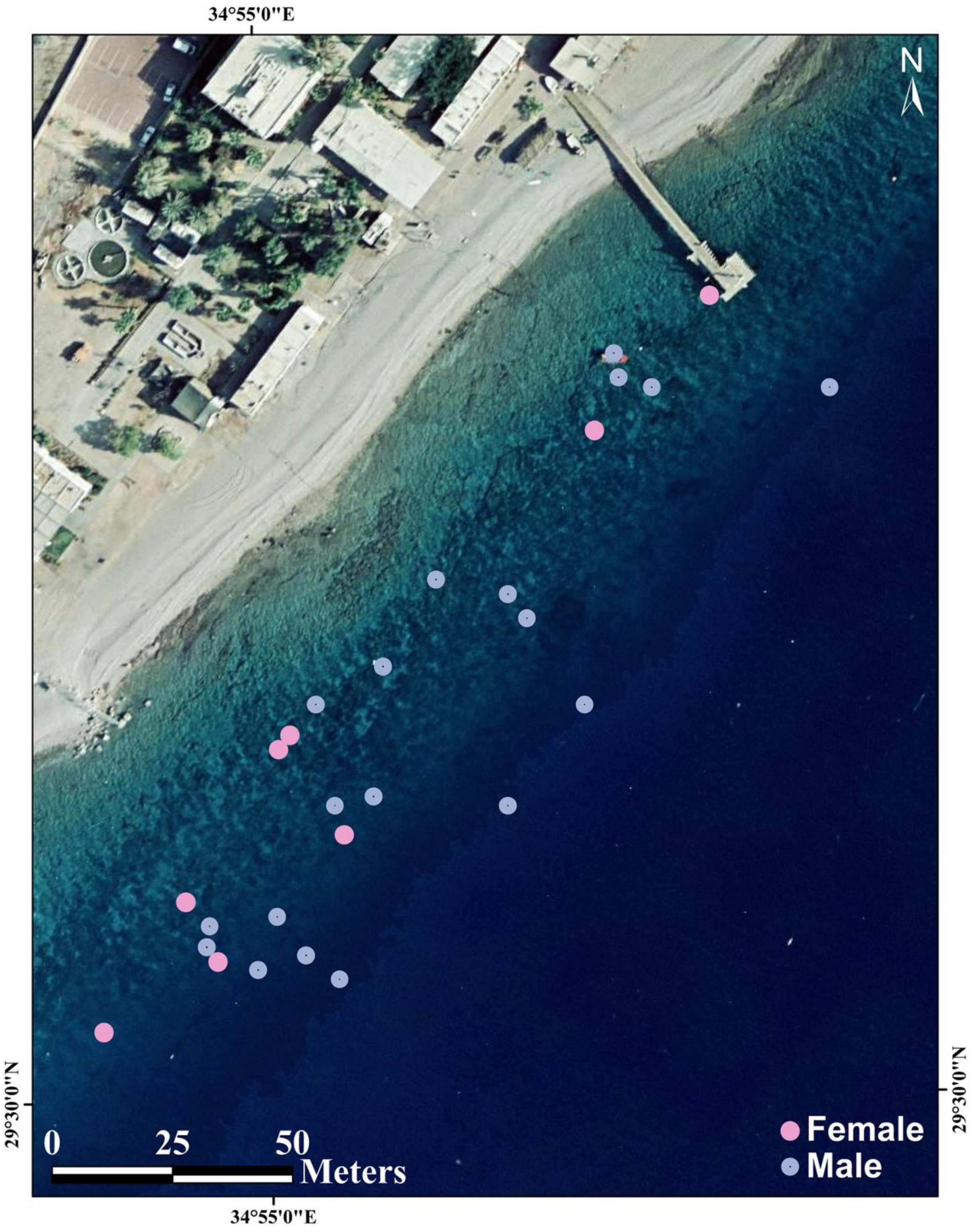

Supplementary Figure S1. An aerial photograph of the IUI site in Eilat (Gulf of Aqaba) showing locations of female (pink dots) and male (blue dots) *Turbinaria reniformis* colonies studied. The photograph, courtesy of Israel Nature and Parks Authority, was edited using ArcGIS V.2.2 (Esri) and Adobe Illustrator CS 6.

Measurements from histological sections of samples from 2003-2009

Supplementary Table S1. Summary of sampling dates of nine tagged *Turbinaria reniformis* colonies in 2003-2009 in Eilat (Gulf of Aqaba) and oocyte measurements from histology. * The measurements from the 27th of June 2008 were combined with measurements from the 22nd of June.

Dates	Sampling		15/3/03	13/7/06	28/8/06	25/5/07	6/7/07	10/4/08	22/6/08	27/6/08	31/7/08	27/8/08	25/9/08	6/11/08	15/3/09	24/5/09	29/7/09
	Full moon			11/7/06	9/8/06		30/7/07		18/6/08	18/6/08	18/7/08	16/8/08					7/7/09
Colony tag	166	Average colony oocyte diameter (μm)	423.51	438.8	115.74	417.51	496.4	397.82	330.92		334.89	80.08	164.99		413.54		378.72
	254		341.12					421.07	414.047		0	86.53	80.89			347	0
	250			0	0	0	0	302.94	364.5717	410.29	0	59.85	71.04	56.01		463.55	0
	246		376.78	434.67		364.29	461.31	0	467.46	0	0	0	100.74			470.88	0
	Number of measured oocytes		30	13	8	20	20	31	24	6	10	27	37	5	11	29	7
	Average oocytes diameter (μm) of all colonies ± SD		380.46 ± 65.51	435.85 ± 151.33	115.74 ± 17.33	390.9 ± 46.8	478.85 ± 59.89	373.97 ± 76.46	389.78 ± 95.46	*	334.89 ± 112.95	78.42 ± 29.91	103.84 ± 43.75	56.01 ± 28.3	413.54 ± 29.71	421.14 ± 80.15	378.72 ± 86.88
	Number of sampled colonies		3	3	2	3	3	4	4	2	4	4	4	1	1	3	4
	% reproductive colonies		100	66.6	50	66.6	66.6	75	100	50	25	75	100	100	100	100	25

Supplementary Video S1. *Turbinaria reniformis* colonies spawning observed at the Eilat Coral Nature Reserve in July 2016
